# Applications of the Capability Approach in the Health Field: A Literature Review

**DOI:** 10.1007/s11205-016-1356-8

**Published:** 2016-05-10

**Authors:** Paul Mark Mitchell, Tracy E. Roberts, Pelham M. Barton, Joanna Coast

**Affiliations:** 1 Health Economics Unit, Institute of Applied Health Research, University of Birmingham, Birmingham, UK; 2 Department of Social Science, Health and Medicine, School of Social Science and Public Policy, King’s College London, London, UK; 3 School of Social and Community Medicine, University of Bristol, Bristol, UK

**Keywords:** Multidimensional poverty, Physical activity, Patient empowerment, ICECAP capability measures, Health functioning

## Abstract

The primary aims of this review are to document capability applications in the health field and to explore the objectives and decision-rules of studies measuring capability more broadly. Relevant studies are identified using a literature search strategy known as “comprehensive pearl growing”. All studies with a primary focus on health are assessed individually, whilst a summary narrative analysis of the full review examines the objectives of capability studies. Four distinct groups in the health field are identified in the review: (1) physical activity and diet; (2) patient empowerment; (3) multidimensional poverty and (4) assessments of health and social care interventions. Different approaches to applying mixed methods, selecting capability dimensions and weighting capabilities are found across studies. There is a noticeable non-reliance on health status as a sole indicator of capability in health. In terms of objectives of studies measuring capability, although there is a lack of consistency, an objective related to sufficiency of capabilities appeared most often in the studies found in this review. Even though one of the appeals of the capability perspective is its underspecified nature, this review highlights the challenge of finding a coherent alternative to more established approaches of evaluation.

## Introduction

The capability approach is a broad normative framework that provides an alternative to welfare economic approaches to evaluating well-being, with a primary focus on individual’s ability to achieve valuable functionings in life (Sen 1993). The capability approach has attracted interest from a wide variety of researchers, scholars and policymakers alike, with the Human Development and Capability Association consisting of 15 thematic groups in May 2016. A number of literature reviews on empirical applications *across* disciplines have been conducted, relating to the aspects of the capability approach that are the focus for analysis (Kuklys and Robeyns 2005), the spread of capability applications across disciplines (Robeyns 2006) and the types of statistical approaches taken when measuring capability (Chiappero-Martinetti and Roche 2009). A number of researchers have also attempted to conceptualise the capability approach for health specifically (Law and Widdows 2008; Ruger 2010; Venkatapuram 2011; Entwistle and Watt 2013). The primary aims of this review are to document studies that apply the capability approach in the health field and to explore the objectives and decision-rules (i.e. criteria for deciding to accept or reject proposal) of studies measuring capability more broadly.

Health itself is notoriously difficult to define (Venkatapuram 2013) and we do not attempt that task here, focusing more on how authors have conceptualised their own study. In terms of practical application of the capability approach within the health field, however, less is known about the breadth and scope of studies being undertaken. Three reviews have assessed the development of capability measures in health economics (Lorgelly et al. 2010; Coast et al. 2015; Lorgelly 2015). However, it is less clear what the current evidence base is on topics related to health in general. The first aim of this study is to document studies that apply the capability approach in the health field.

Less attention has also been paid to how capability measures are being used to inform policy making. Although there are widely documented indices informed by the capability approach [for example, the Human Development Index (HDI), the Multidimensional Poverty Index (MPI)], these are focused mainly on international comparisons; less is known about the purpose or objectives of capability applications across other fields of research. Given the underspecified nature of the capability approach (Gasper 2007), it would be informative to know how capability objectives are interpreted for particular policy areas across different fields. The second aim of this paper is to establish the analytical objectives when applying measures of capability. Since little has been written on this specific topic within the health field, in achieving this second aim, the research draws on the literature across all fields.

These aims will be achieved through undertaking a literature review, applying a search strategy known as “comprehensive pearl growing” (Hartley et al. 1990) to identify studies measuring capability with a primary focus on health. Then, a summary narrative analysis of studies included in the review across other fields will be used to detail how researchers, in general, are applying the capability approach to aid decision-making.

The remainder of the paper is structured as follows. Section 2 details the comprehensive pearl growing search strategy employed to identify relevant studies. The method of identifying studies through key ‘pearls’ and inclusion and exclusion criteria used in the study are then explained. Data extraction and methods of analysis used are given before summary results of the literature search and grouping of papers by themes is presented. Given the primary focus of the review on the topic of health, the literature relating to health is then reviewed in much greater detail. Studies in the broad area of health are first grouped and described according to four key sub-themes. They are then compared in terms of the methodologies that they use, particularly in relation to how capabilities are selected and how weights are assigned to the selected capabilities. A summary narrative analysis of objectives and decision-rules across all other studies outside the health field is then presented. A discussion of the literature review findings concludes the paper.

## Methods

### Literature Search Strategy

The search strategy employed in this review is known as “comprehensive pearl growing”, a particularly useful search strategy for interdisciplinary topics (Schlosser et al. 2006). The process of pearl growing commences with the identification of ‘key pearls’ (i.e. key studies), that can be identified from within the literature as being compatible with the aim of the review (Hartley et al. 1990). Once the key pearls have been identified, these are used to generate the ‘first wave of pearls’, that is, papers that have cited the key pearls within their reference list. Essentially, this type of search uses forward citations emanating initially from ‘key pearls’ and then from subsequent waves of pearls.

The literature search was undertaken through the Institute of Scientific Information (ISI) Web of Knowledge citation search online facility. The ISI Web of Knowledge covers a number of databases including Web of Science (sciences, social sciences, arts and humanities) and MEDLINE (biomedicine and health sciences), which made it an appropriate database for searching capability literature across a wide variety of disciplines.

Papers published between 1 January 2006 and 1 December 2012 were included in the initial search, with the review updated to include studies on ISI until 1 November 2014. The review aimed to focus on the most recent advances in the operationalisation of the capability approach, given that earlier studies were already likely to have been captured through previous capability empirical reviews (Kuklys and Robeyns 2005; Robeyns 2006; Chiappero-Martinetti and Roche 2009).

### Selection of Key Pearls

To identify the key pearls, research included in three previous reviews was considered (Kuklys and Robeyns 2005; Robeyns 2006; Chiappero-Martinetti and Roche 2009). The broadest disciplinary focus from the previous reviews was the study by Robeyns (2006) who identified nine areas where the capability approach has been applied (Robeyns 2006). Two groups (critiquing social norms and non-normative research) were excluded as the studies were not relevant for the focus of this review. From each of Robeyns’ remaining seven groups, at least one study per group was chosen as an initial key pearl. Nine ‘key pearls’ from Robeyns’ (2006) review were included and the Robeyns review (2006) itself was the tenth key pearl. Overall ten key pearls were included (Chiappero-Martinetti 2000; Alkire 2002; Fukuda-Parr 2003; Robeyns 2003; Ruggeri Laderchi et al. 2003; Kuklys 2005; Lewis and Giullari 2005; Zaidi and Burchardt 2005; Anand and van Hees 2006; Robeyns 2006).

### Inclusion Criteria and Paper Categorisation

To be included, studies required to be published papers in English and needed to address at least one of the two review objectives:

1. the aggregation of capability at an individual level (i.e. domains of capability included) and/or across populations (i.e. how capability between individuals are compared), and/or
2. an objective or decision-rule as to how such outcomes could be then used to aid decision-making

Based on the above criteria, titles and abstracts for the studies were sorted through keyword searching. Keyword searching through title and abstract was structured as follows:

- Capability OR Capabilities OR Functioning(s) OR Agency [Capability keyword]
- AND
- Measure OR Outcome OR Empirical OR Index OR Operationalisation [Measurement keyword]

Studies excluded are non-English publications, books and book chapters, conference abstracts and presentations. Additionally, studies conducting validity of capability measures in certain patient groups were excluded as they were beyond the scope of this review.

This review followed a two stage process of study categorisation. This follows from previous reviews that have used this categorisation process to identify the studies of most relevance to the research question at hand (Roberts et al. 2002).

#### Stage I: Initial Categorisation of Studies

The studies identified using the previously outlined search strategy were then sorted into three categories based on the title and abstract.

Category A: studies that mentioned at least one capability keyword AND at least one measurement keyword.

Category B: studies that could be potentially relevant to the review, but required more information than the title and abstract alone. If the study contained at least one capability keyword but no measurement keyword, the study was examined for a results section including tables and figures, which could potentially indicate an attempt to measure capability outcomes. If a measurement keyword was found in the title and abstract but no capability keyword, the reference list for the study was searched for citations of key capability writings by either Amartya Sen (Sen 1985, 1992, 1993, 2000, 2009) or Martha Nussbaum (Nussbaum 2000, 2011), as a means of eliciting whether the study was concerned with capability.

Category C: studies that were excluded from the review. The studies either did not include any of the capability or measurement keywords or did not meet the criteria for Category B.

**Fig. 1:**
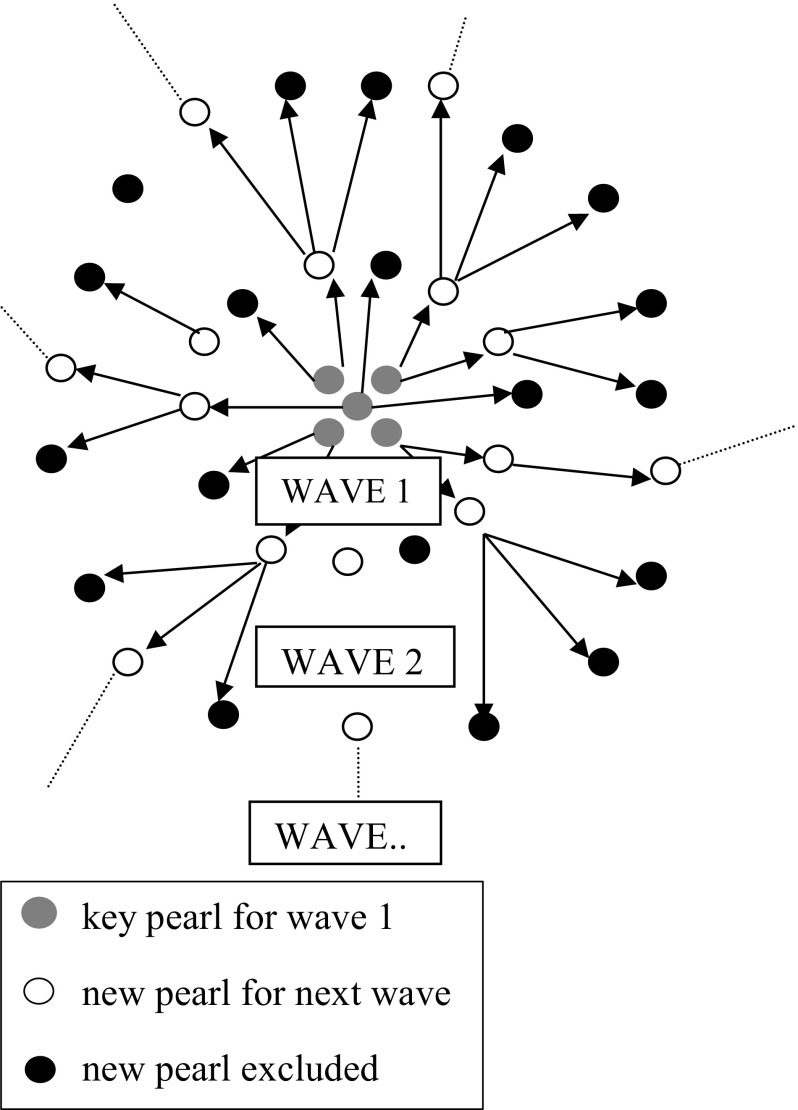
Illustration of the comprehensive pearl growing method

Studies identified from the first wave that were categorised as Category A or B were then employed to carry out a further wave search. Studies that had cited these new pearls were then categorised in the same manner as in the first wave. This process of wave searching continued until no new relevant studies were found. An illustration of the pearl growing method is presented in Fig. 1.

#### Stage II: Further Classification of Studies

Following the completion of the pearl search, studies categorised as either A or B were further classified after being read in full. Four classifications were used to identify the final papers for inclusion.

1. Study developed and/or applied capability related outcome and discusses decision rule
2. Study developed and/or applied capability related outcome but does *not* discuss decision rule
3. Study discusses decision rule but does *not* develop nor apply capability related outcome
4. Study does not develop nor apply capability related outcome nor discuss decision rule

Papers within the first three classifications were included in the analysis. Papers within the fourth classification were excluded from further analysis.

The review update used all studies classified within the first three categories during the initial search as the starting ‘key pearls’ for the update.

### Data Extraction

Data were extracted from all included studies using a standardised data extraction form. This follows protocol for data extraction from systematic reviews in health (CRD 2009). Information extracted from studies include name of author(s); title of study; year of publication; dimensions within capability related measure; thematic group study most associated with; context capability measure developed for; country study conducted; study country specific or multinational; whether comparisons are made between different population groups; objective of study; and what decision criteria/rules discussed (see Appendix 1). The initial extraction and thematic grouping was undertaken by the first author and co-authors checked that the grouping of studies by theme most accurately reflected the primary focus of each individual study.

### Analysis

Robeyns’ (2006) seven application groups (i. General assessment of human development; ii. Assessing small scale development projects; iii. Identifying the poor in developing countries; iv. Poverty and well-being assessment in advanced economics; v. Deprivation of disabled people; vi. assessing gender inequalities; vii. Debating policies) that identified key pearls for this review are used as a starting point for grouping and analysing all the studies included following the pearl search; where studies fell outside these groups, additional groups for new themes were generated when necessary. Those grouped in a health thematic group are of primary interest in this review and we detail each study found, as well as comparing and contrasting the studies found in this group. To explore objective and decision-rules across fields outside of health, a narrative summary of the studies found in the remainder of the review is presented to give an overview of approaches across thematic groups.

## Results

### Summary of Pearl Search

**Fig. 2:**
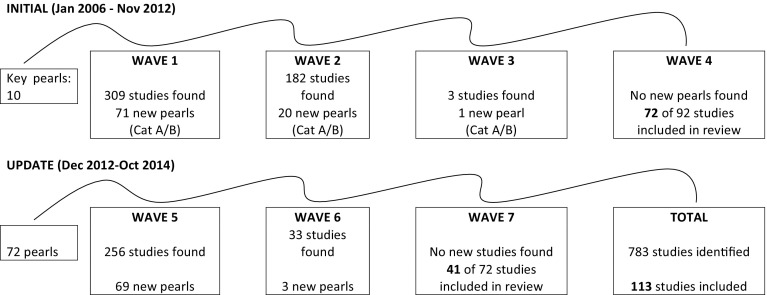
Summary statistics of initial comprehensive pearl growing review results

The summary of the literature search is provided in Fig. 2. Out of 783 studies identified in the seven waves of literature searches, 113 studies are included in the review (see Appendix 2).

### Summary Data for Studies Included

**Fig. 3:**
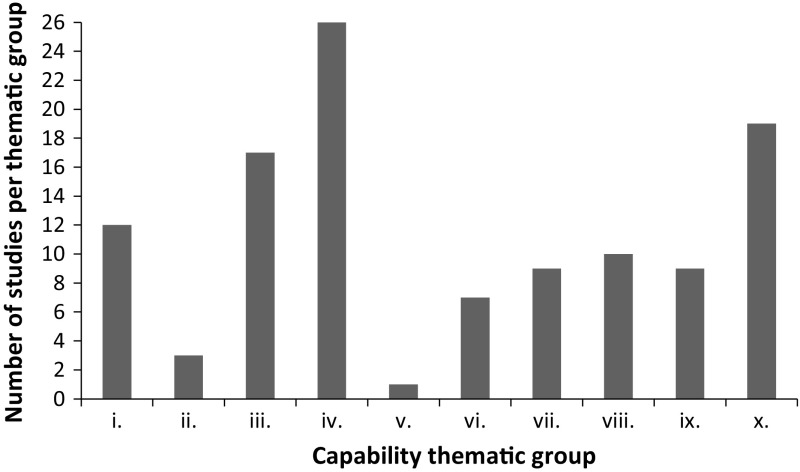
Number of studies per capability thematic group. Capability thematic groups: *i* general assessment of human development; *ii* assessing small; scale development projects: *iii* identifying the poor in developing countries, *iv* poverty; well-being assessment in advanced economics: *v* deprivation of disabled people, *vi* assessing gender inequalities: *vii* debating policies, *viii* education, *ix* technology, *x* health

Figure 3 shows the spread of studies across seven capability thematic groups identified by Robeyns (2006) (i.e. group i.–group vii.) and three new themes that emerged from this review (i.e. group viii.–group x.). Group iv. (assessing poverty and well-being assessment in advanced economies) has the highest proportion of studies identified out of the 10 groups with 26 studies. The three new groups, education (group viii), technology (group ix.) and health (group x.), account for 37 of the 113 studies identified, showing a growing interest in capability applications in these three groups in particular. Indeed, the health group produced the second largest number of studies, with 19 papers focused primarily in the health field.

### Thematic Group x. Health

The health thematic group consisted of nineteen studies in total and are first detailed in four sections, before a comparison of studies is presented.

#### Physical Activity

Four studies from the United States looked into how the capability approach could be applied in assessing people’s capability to engage in physical activity with the aim of improving their health. Lewis (2012a, b) undertook two studies looking into how the built environment in communities can be a leading instigator into the capability of individuals to participate in physical activity, exploring what questionnaires would be appropriate to capture this capability (Lewis 2012a, b). Ferrer also led two studies, tackling problems associated with lack of physical activity in different ways. The first, Ferrer and Carrasco (2010), developed a list of capabilities to assess patient’s ability to manage their own health behaviours through an 18 item list on diet and physical activity (Ferrer and Carrasco 2010). The second and more recent, Ferrer et al. (2014) developed an instrument on healthy diet and physical activity using a more rigorous mixed methods approach. Qualitative focus groups were conducted with members from the Latino community, who had obesity and diabetes, to ascertain the constraints on opportunities to pursue healthy behaviour. From the focus groups, eight scales measuring capability approach constructs were produced: two subscales for resources and six subscales on conversion factors. The authors emphasise that their results show the need to focus on practical opportunities for healthy behaviour that will help bridge goals of intention with achievement of a healthy lifestyle (Ferrer et al. 2014).

#### Empowerment in Health

Three studies identified were concerned with patient empowerment. Two studies identified in the review worked on measuring women’s empowerment in developing countries when it came to decision-making relating to their health. Mabsout (2011) developed a health functioning model for Ethiopian women, with the aim to reduce shortfalls in health through measuring education, earnings share, control over earnings and decision-making (Mabsout 2011). Nikiema et al. (2012) pursued a similar theme by assessing women’s perceived ability to access healthcare in Burkina Faso by knowing where to go to seek care, getting permission to go, getting money for treatment, distance to health facility, having to take transportation, not wanting to go alone and concern that there may not be a female healthcare provider available. The third discussed the trade-offs associated with patient empowerment versus the maximisation of a patient’s health status (McAllister et al. 2012).

#### Multidimensional Poverty in Health Groups

Three studies were concerned with assessing multidimensional poverty in a health setting. Callander et al. (2013a, b) developed what they termed as a Freedom Poverty Measure, assessing poverty in terms of three dimensions: income, education and health. Although similar in make-up to the HDI, Callander et al. (2013a) placed additional weight on income due to its perceived importance in assessing poverty in Australia. Using a national representative survey from the Australian Bureau of Statistics in 2003, of those classified as multidimensionally poor, three out of four had a chronic health condition. Having a chronic condition meant individuals were three times more likely to be multidimensionally poor, raising to seven times as likely if the chronic health condition was depression/mood affecting disorder (Callander et al. 2013a). Callander et al. (2013b) also used the same dataset and applied their freedom poverty measure to assess freedom poverty in people with cardiovascular disease.

Mitra et al. (2013) used a mixed methods approach to develop a multidimensional poverty measure to assess the situation of persons with psychiatric disorders in the United States. First, Mitra and colleagues developed a theoretical capability list before focusing on seven empirical capabilities that could be measured through the Medical Expenditure Panel Survey. These empirical capabilities were then the subject of two focus groups who were asked to rank the importance of each capability. Rankings based on a lived experience group of psychiatric disorders and a providers/research group were used to weight capabilities separately, and apply two different methods of weighting the capabilities based on their ranking (Mitra et al. 2013). Results showed that the measure of multidimensional poverty was sensitive to the selection of different ranking and weighting structures. Mitra et al. (2013) concluded that further development of multidimensional measures should proceed using larger scale qualitative methods or combined methods (i.e. qualitative and quantitative) when ranking and weighting the capabilities (Mitra et al. 2013).

#### Assessments of Health and Social Care Interventions

Nine studies developed and/or applied capability measures for assessing health and care interventions for different population groups. Of these nine, six studies were concerned with the development of the ICEpop CAPability measure for Older people (ICECAP-O). Using qualitative interviews with older adults in the United Kingdom, it was found that the capability to achieve important functionings was of primary interest to this population (Grewal et al. 2006). Subsequently, thematic analysis was undertaken on the qualitative interviews to develop a short self-complete questionnaire (ICECAP-O) capturing the most important capabilities for this age group (Coast et al. 2008). Five capabilities were found to be of primary importance: attachment, role, enjoyment, security and control (Coast et al. 2008). The five capabilities were then assigned weights to create an index, based on a random utility methodology known as best-worst scaling (Coast et al. 2008). The ICECAP-O has now been used to assess capability in a number of different country and health settings, such as in fall patients in Canada (Davis et al. 2013), arthritis patients in the UK (Mitchell et al. 2013), older carers in Australia (Ratcliffe et al. 2013) and the general population for public health research in Australia (Couzner et al. 2013). A similar, although distinct, measure for the general adult population is the ICECAP-A, which has been developed using the same methodology. It also has five attributes of capability, although in the general adult population the most important attributes are stability, attachment, achievement, autonomy and enjoyment (Al-Janabi et al. 2012).

Another capability measure aimed primarily at older adults is the adult social care outcomes toolkit (ASCOT) (Netten et al. 2012). Also using qualitative methods to develop attributes, Netten et al. (2012) found nine areas to include when assessing social care interventions: food and drink, personal care, safety, social participation and involvement, control over daily living, accommodation, cleanliness and comfort, occupation and dignity. Although the ASCOT was originally developed without theoretical justification from the capability approach, the most recent ASCOT has an emphasis on the newly developed highest level of each attribute on the wants and likes of social care users to reflect a broader aspect of the questionnaire on capability. The lower three levels on the ASCOT reflect levels of basic functioning (Netten et al. 2012). Using preference weighting to develop a measure of social care related quality of life, Netten et al. (2012) developed a measure that can be used to produce a social care quality adjusted life year (QALY), allowing comparisons with the health QALY to judge resource allocation across health and social care interventions. QALYs are used by health guidance bodies, such as the National Institute for Health and Care Excellence (NICE) in England, to assess the cost-effectiveness of interventions based on their contribution to morbidity and mortality improvements (NICE 2014).

A final study developed a capability questionnaire for assessing the capability of mental health patients (Simon et al. 2013). Rooted in Nussbaum’s list of 10 essential human capabilities (Nussbaum 2000), the Oxford Capability Mental Health (OxCap-MH) measure consists of 18 items that has been developed from previous attempts to formulate Nussbaum’s list into a questionnaire format (Simon et al. 2013). Simon et al. (2013) tested their capability instrument within the Oxford Community Treatment Evaluation Trial and developed a capability index. In terms of valuing capability items, each item is weighted equally.

### Comparison of Studies in the Health Group

There are a variety of ways the capability approach is being adopted in health and the individuals who are targeted by researchers measuring capabilities vary across studies. A mixed methods approach appears popular across a large number of the studies. However, there are key distinctions as to when qualitative and quantitative methods are applied. For Mitra et al. (2013), key aspects of capability for their population of interest are first identified through their research team, before asking focus groups the importance of the capability indicators they present. Simon et al. (2013) adopt a similar approach using Nussbaum’s list of 10 essential capabilities as their basis of capability indicators to include before testing their questionnaire in a mental health population. Alternatively, other studies have first asked their population of interest what aspects of life are important to them: obese or diabetic (Ferrer et al. 2014), people 65 years and older (Grewal et al. 2006), and social care users (Netten et al. 2012). For people 65 years and older, this led to a capability questionnaire being developed through thematic analysis by the research team (Coast et al. 2008). Ferrer et al. (2014) adopted a quantitative approach to finalising their questionnaire, using principal component analysis to generate a measure capturing resources and conversion factors to engage in a healthy diet and physical activity. For social care users, a previous version of a social care questionnaire was modified to reflect what was found in qualitative interviews and to attempt to capture capability (Netten et al. 2012).

A number of different weights across dimensions were applied. Simon et al. (2013) followed Nussbaum’s stance on the importance of achieving all capabilities in their list, so equal weight was attached to the 10 dimensions, although this approach indirectly gives double weight to dimensions that have two questions per dimension compared to one question per dimension. Callander et al. (2013a, b) give additional weight to income in their freedom poverty measure of education, health and income; an unusual approach in capability literature where focus has shifted on moving away from income in a multidimensional poverty space (Alkire and Foster 2011). To be classified in freedom poverty, meaning multidimensionally poor, individuals needed low income (below 50 % of median income poverty line) *and either* poor overall health status (lower than average SF-6D utility score for their age group) *or* have insufficient education (less than 12 years aged 25–64 or less than 10 years aged 65 and above) (Callander et al. 2013a). Mitra et al. (2013) use quantitative weighting formulas based on the ranking of importance of dimensions found in their focus groups. Netten et al. (2012) weight their social care instrument using a mixture of time trade-off and best-worst scaling methodology. These methods, in particular time trade-off are most prominently used to weight health states in QALYs for health economic evaluations. Coast et al. (2008) have argued against the use of the preference-based time trade-off methodology when weighting capabilities. Instead, Coast et al. (2008) base capability weighting on best-worst scaling only, arguing this approach involves population value judgements as opposed to people’s preferences.

What is noticeable in all studies is the lack of reliance on health status as the sole measure of capability, suggesting a shift in the evaluative space from functionings to capability in the studies found in this review. For example, Mabsout (2011) and Nikiema et al. (2012) find that focusing on women’s ability to make decisions with regard to health seeking behaviour takes prominence. McAllister et al. (2012) discusses how there could be a tradeoff between maximising health in favour of patient empowerment, although they do not detail how this may work in practice.

Across the 19 studies in the health group, there does not appear to be a sole objective reflective of the capability approach. Netten et al. (2012) take the traditional health economics route of implementing a measure using preference weights to develop a social care QALY to aid social care decision-making in terms of cost-effectiveness, with the aim of maximising QALY gains. However, such an approach is outright rejected by Simon et al. (2013), who argue that incorporating preferences similar to the QALY approach goes against the underlying rationale of the capability approach.

### Capability Objectives and Decision-Rules

This section provides a narrative summary of the objectives and decision-rules from the studies identified in this review not classed in the health group (see Appendix 2).

Although the capability approach was developed as an alternative to the traditional utilitarian approach in welfare economic assessment, there are some studies identified in this review who argue that capabilities can fall within a similar maximisation framework. One example of this is Renouard (2011), whose study suggests that corporate social responsibility within private enterprise should account for what they term as “relational capability”. By drawing upon research within anthropology and Sen and Nussbaum’s research, Renouard (2011) proposes to look beyond utility maximisation of company stakeholders but rather achieve the:

maximisation of the relational capability of people impacted by the activities of companies (Renouard 2011)

This concept of maximising an absolute level of capabilities is not limited to the above example, with Biggeri and Ferrannini suggesting an objective of “maximising freedoms” in development analysis (Biggeri and Ferrannini 2014). Tikly and Barrett (2011) also state that the capability approach of “maximising choice” is a more appropriate assessment of welfare than the standard rational choice theory of economics within education of low income countries:

Here the assumption is that individuals act on the basis of the maximisation of their own utility and that efficiency within the public welfare system is best served through maximising ‘choice’ (Tikly and Barrett 2011)

However, the objective of maximising capabilities in some form or another as an absolute aim is not a reflection of the majority of work related to the papers found in this review. As an example of this, Anand et al. (2009) states:

they (people) do not wish to maximize total social welfare for a variety of reasons, not least of which is that they are concerned about distributional issues too (Anand et al. 2009)

Many papers focus on the maximisation of something less than optimum levels as a priority, such as the maximisation of basic capabilities (Krishnakumar and Ballon 2008) or by measuring poverty as “insufficiency in basic capabilities” (Kerstenetzky and Santos 2009).

Other conceptualisations of the capability approach have developed within more advanced economies. Binder and Broekel (2011) develop their concept of “conversion efficiency” as an alternative to traditional well-being assessment:

The idea of relative efficiency means we are evaluating individuals’ efficiency not with a theoretically derived maximum, but to the maximum of functioning achievement observed in the data given a certain level of resources (Binder and Broekel 2011)

Binder and Broekel (2011) demonstrated their measure within Great Britain and showed that conversion efficiency is improved within this sample, by age, self-employment, marriage, the absence of any health problem and living in London and the surrounding boroughs.

Murphy and Gardoni (2010) developed a two-stage process for assessing individual capability within a risk analysis, such that:

for defined groups, the goal should be to maximise variability of non-basic capabilities and minimise variability within sub-vectors of basic capabilities and among defined groups of those with similar boundary conditions (Murphy and Gardoni 2010)

Another alternative to welfare maximisation in a narrow space comes from the field of education. Callander et al. (2012) argue that increasing educational opportunities for youths is not an adequate pre-requisite to future labour force participation. Instead they develop a measure drawn from the multidimensional poverty literature (Alkire and Foster 2011) to assess health alongside education, which they argue is also likely to have an impact of the probability of labour force participation in the future:

efforts to increase children’s future labour participation rates as a means of improving their living standards should also focus on improving childhood health, as well as education. (Callander et al. 2012)

From this review, there does not appear to be a method for combining a measure of capability with the cost of an intervention, even though studies have developed outcomes as alternatives to measuring benefits monetarily in a cost-benefit analysis (Beyazit 2010; Gardoni and Murphy 2010).

## Discussion

This study provides an up to date review of empirical capability applications, focusing particularly on publications interested in measuring capability in health and aiding decision-making more generally. Through an overview and comparison of research in the health field and a summary narrative analysis of studies across identified thematic groups, the review finds a number of different interpretations as to what capabilities to focus on depending on the intervention under consideration. Although this can be argued to be one of the benefits of the capability approach, drawing from the “toolbox” to suit a given research question (Conradie and Robeyns 2013), it also leads to practical difficulty. Mitra et al. (2013) summarise this problem neatly:

Although there is conceptual value to its voluntary incompleteness, Sen’s approach makes the capability approach difficult to operationalize (Mitra et al. 2013)

This study provides an overview as to how researchers are attempting to measure capability and inform decision and policy making, particularly in the health field. The health overview provides information on how different research groups are drawing from the same approach to solve similar problems in different ways, albeit with the caveat that different individuals are targeted for analysis by different researchers. The narrative analysis of objectives within capability empirical applications provides an overview as to how researchers across disciplines are using the approach to address policy needs, where we try to determine the level of consistency across a wide variety of subject fields as to what is the primary objective when measuring capability in practice.

The capability approach continues to grow in its application, with this review requiring the classification of three new capability thematic groups compared to a previously conducted review (Robeyns 2006). Although this is an encouraging development, it could lead to a lack of coherence in utilising the capability approach even in the same topic area. Health is a prime example. Four different sections were required to describe the analysis using a capability approach in the same, albeit vast, field. Not only is the capability approach being applied in different ways, researchers have different interpretations of what it means to employ a capability perspective. However, the focus in all studies in the health field in moving away from a reliance on health status alone towards capturing individual capability provides a form of agreement in this area. This compares to a previous review of capability empirical applications across disciplines that found researchers using the capability approach most commonly measured functioning attainment (e.g. good health) rather than attempting to capture the capability to attain such functionings (Robeyns 2006). This indicates that studies within the health field are rising to the challenge of the “capability criterion”, i.e. measuring the opportunity aspect of the capability approach that makes it a distinct framework from traditional evaluative approaches, such as welfare economics (Gasper 2007).

The primary results show that there is a pluralistic interpretation as to how the capability approach should be applied either in health or elsewhere to aid decision-making. Therefore, decision-makers who are used to a consistent approach may struggle to apply the capability perspective in practice if there is no guiding principle as to what a capability based evaluation should look like and what is its overall objective. However, the majority of the studies reviewed across thematic groups appear to follow a trend related to achieving “basic capabilities” (Young 2009) or a “minimum level of capabilities attainment” (Murphy and Gardoni 2008). This threshold approach has also been referred to within regions as a “sufficiency economy” (Parks 2012) or within adult literacy as a “sufficient” level of learning (Maddox and Esposito 2011). Although there may be some crossover with a sufficiency objective and conceptualisations of the capability approach for health, it is not clear that the leading conceptual authors of the capability approach for health (Ruger 2010; Venkatapuram 2011) or more generally (Nussbaum 2011; Sen 2009) would support such an objective as an overarching goal.

Compared to similar reviews conducted on capability applications, this study produces a novel way of searching for capability literature in a comprehensive manner, using an explicit methodology. Previous reviews of capability applications did not document how their studies were collated, meaning other researchers could not replicate their search if they so wished. The pearl searching method employed here is also useful for researchers trying to gather studies related to their work and are only aware of a few key publications. There is precedent for using this methodology when searching the health literature for topics where search terms have a number of meanings and relevant papers are not easily identified from non-relevant papers using traditional systematic search strategies (Dolan et al. 2005; Tsuchiya and Dolan 2005; Stafinski et al. 2010, 2011). A review of empirical applications of the capability approach seems similarly well suited to this method, as using a key word search strategy for a term such as “capability” that has a number of different meanings would return thousands of irrelevant studies. This pearl searching process provides a method as to how related studies can be easily identified. Given the spread of application of the capability approach across thematic groups found in this review (see Fig. 3), the need for a comprehensive approach for reviewing literature is more important, as it is unlikely any researcher will be able to identify all related work in their field without using such a comprehensive literature search strategy approach.

There are a number of limitations with this review. The literature search was restricted to published papers in English only. Whilst we are aware that some of work on capabilities often appears in books and other languages, this was a limitation that was necessary, as there was no consistent method for checking whether book chapters or non-English studies were relevant for this review. It is also important to note that this review does not cover the totality of research in the health field or other thematic areas, as it was focused on a particular review question. The studies that met the inclusion criteria for the review tended to be quantitative in nature, given the research focus; it is important therefore not to interpret the findings and selection of studies in this review as being representative of the entirety of research effort concerning the capability approach. Also, as we did not conduct any form of quality check on the papers, ensuring only peer review publications were considered provided some form of quality check.

Although there are positives for the pearl growing search strategy, there are also some negatives. The freedom for the researcher to include any research they wish in their review was not followed here. Additionally, research that fell outside the review search criteria (i.e. year of publication, publication type etc.) is automatically excluded, so relevant studies that are known to the authors of this review that could have been included in this review were not possible (Ibrahim and Tiwari 2014; Greco et al. 2015; Kinghorn 2015; Kinghorn et al. 2015; Lorgelly et al. 2015; Mitchell et al. 2015a, b; Ruger 2015). A more flexible approach of reviewing literature in a comprehensive search may be more appropriate when reviewing capability literature in future. Moreover, the search strategy allowed measures developed closer to the start of the literature search start date (e.g. ICECAP-O) to accumulate other studies using them in practice, compared to more recently developed measures (e.g. ASCOT, ICECAP-A, OxCAP-MH). In addition, future literature reviews related to this topic should also consider qualitative research in the health field, the kinds of questions asked in relevant studies and how the concept of capability relates to the types of questions asked by different researchers.

Our focus in this review was to provide clarity as to how capabilities were measured in the health field and whether or not there is a clear objective when measuring capability. We have shown a wide variety of applications of empirical studies within health and different objectives across disciplines. For clinicians and policymakers to take the capability approach seriously as a viable alternative to the welfare economics influenced approaches that have now been established in evidence based medicine (for example, the incremental cost effectiveness ratio when using the QALY in health economic evaluations), more clarity about alternative objectives is required. Such alternative objectives need to be just as applicable to the health context as to that of poverty assessment if they are to obtain wider usage. Given there is a lack of consistency about how the measurement of capabilities can be used to aid decision-making, the challenge remains as to whether the capability approach can offer a coherent alternative to welfarist or other non-welfarist economic assessments across health and public policy.

## Appendix 1: Data Extraction Sheet

**Table:**

Criteria	Justification
Name of author(s), title of study, year of publication	Summary information necessary for descriptive statistics
Are details available on the type of the attributes within the capability related measure?	Understanding the components of capability related measures across discipline
Type of application of the capability approach? For example poverty and well-being assessment in advanced economies	The Robeyns’ (2006) groups of studies where the capability approach has been applied should help to analyse similar studies together
Was the capability related measure developed for a specific context? If so, which context?	It has been argued by those who have applied of the capability approach that measures can be developed to address a specific policy question
Country study conducted	Can the study findings be applied in a UK setting?
Was the study country/area specific or cross-national/disciplinary? Which country and what area of focus?	It is important to ascertain the potential for interdisciplinary research, as areas which are applied within a number of fields/countries, may be more adaptable to a health analysis setting
Are comparisons made between different population groups?	An important role in allocating resources is the commensurate nature of population comparisons
Objective of study?	Health maximisation, poverty reduction etc..
Are decision criteria/rules discussed? What methods were used?	If a measure has been promoted within a study, do the authors suggest how decision-makers should interpret such results for aiding decision-making?

## Appendix 2: Studies Included in Final Review Across Capability Thematic Groups

### Group i. General Assessment of Human Development

Alkire, Sabina, and James Foster. 2011. “Counting and multidimensional poverty measurement.” *Journal of Public Economics* no. 95 (7–8):476–487. doi: 10.1016/j.jpubeco.2010.11.006.

Alkire, Sabina, Ruth Meinzen-Dick, Amber Peterman, Agnes Quisumbing, Greg Seymour, and Ana Vaz. 2013. “The Women’s Empowerment in Agriculture Index.” *World Development* no. 52 (0):71–91. doi: 10.1016/j.worlddev.2013.06.007.

Alkire, Sabina, and Maria Emma Santos. 2014. “Measuring Acute Poverty in the Developing World: Robustness and Scope of the Multidimensional Poverty Index.” *World Development* no. 59 (0):251–274. doi: 10.1016/j.worlddev.2014.01.026.

Alkire, Sabina, and MariaEmma Santos. 2013. “A Multidimensional Approach: Poverty Measurement & Beyond.” *Social Indicators Research* no. 112 (2):239–257. doi: 10.1007/s11205-013-0257-3.

Anand, Paul, Jaya Krishnakumar, and Ngoc Bich Tran. 2011. “Measuring welfare: Latent variable models for happiness and capabilities in the presence of unobservable heterogeneity.” *Journal of Public Economics* no. 95 (3–4):205–215. doi: 10.1016/j.jpubeco.2010.11.007.

Distaso, Alba. 2007. “Well-being and/or quality of life in EU countries through a multidimensional index of sustainability.” *Ecological Economics* no. 64 (1):163–180. doi: 10.1016/j.ecolecon.2007.02.025.

Gardoni, Paolo, and Colleen Murphy. 2010. “Gauging the societal impacts of natural disasters using a capability approach.” *Disasters* no. 34 (3):619–636. doi: 10.1111/j.1467-7717.2010.01160.x.

Krishnakumar, Jaya, and Paola Ballon. 2008. “Estimating Basic Capabilities: A Structural Equation Model Applied to Bolivia.” *World Development* no. 36 (6):992–1010. doi: 10.1016/j.worlddev.2007.10.006.

Nguefack‐Tsague, Georges, Stephan Klasen, and Walter Zucchini. 2011. “On Weighting the Components of the Human Development Index: A Statistical Justification.” *Journal of Human Development and Capabilities* no. 12 (2):183–202. doi: 10.1080/19452829.2011.571077.

Notten, Geranda, and Keetie Roelen. 2012. “A New Tool for Monitoring (Child) Poverty: Measures of Cumulative Deprivation.” *Child Indicators Research* no. 5 (2):335–355. doi: 10.1007/s12187-011-9130-6.

Nussbaumer, Patrick, Morgan Bazilian, and Vijay Modi. 2012. “Measuring energy poverty: Focusing on what matters.” *Renewable and Sustainable Energy Reviews* no. 16 (1):231–243. doi: 10.1016/j.rser.2011.07.150.

Rende, Sevinc, and Murat Donduran. 2013. “Neighborhoods in Development: Human Development Index and Self-organizing Maps.” *Social Indicators Research* no. 110 (2):721–734. doi: 10.1007/s11205-011-9955-x.

### Group ii. Assessing Small Scale Development Projects

Biggeri, Mario, and Andrea Ferrannini. 2014. “Opportunity Gap Analysis: Procedures and Methods for Applying the Capability Approach in Development Initiatives.” *Journal of Human Development and Capabilities* no. 15 (1):60–78. doi: 10.1080/19452829.2013.837036.

Conradie, Ina, and Ingrid Robeyns. 2013. “Aspirations and Human Development Interventions.” *Journal of Human Development and Capabilities* no. 14 (4):559–580. doi: 10.1080/19452829.2013.827637.

Peris, Jordi, Sarai Fariñas, Estela López, and Alejandra Boni. 2012. “Expanding collective agency in rural indigenous communities in Guatemala: a case for El Almanario approach.” *International Development Planning Review* no. 34 (1):83–102. doi: 10.3828/idpr.2012.6.

### Group iii. Identifying the Poor in Developing Countries

Alkire, Sabina, and Suman Seth. 2013. “Selecting a Targeting Method to Identify BPL Households in India.” *Social Indicators Research* no. 112 (2):417–446. doi: 10.1007/s11205-013-0254-6.

Ansari, Shahzad, Kamal Munir, and Tricia Gregg. 2012. “Impact at the ‘Bottom of the Pyramid’: The Role of Social Capital in Capability Development and Community Empowerment.” *Journal of Management Studies* no. 49 (4):813–842. doi: 10.1111/j.1467-6486.2012.01042.x.

Arndt, Channing, Andres Garcia, Finn Tarp, and James Thurlow. 2012. “Poverty Reduction and Economic Structure: Comparative Path Analysis for Mozambique and Vietnam.” *Review of Income and Wealth* no. 58 (4):742–763. doi: 10.1111/j.1475-4991.2011.00474.x.

Barrientos, Armando. 2010. “Protecting Capability, Eradicating Extreme Poverty: Chile Solidario and the Future of Social Protection.” *Journal of Human Development and Capabilities* no. 11 (4):579–597. doi: 10.1080/19452829.2010.520926.

Batana, YéléMaweki. 2013. “Multidimensional Measurement of Poverty Among Women in Sub-Saharan Africa.” *Social Indicators Research* no. 112 (2):337–362. doi: 10.1007/s11205-013-0251-9.

Clark, David A., and Mozaffar Qizilbash. 2008. “Core Poverty, Vagueness and Adaptation: A New Methodology and Some Results for South Africa.” *The Journal of Development Studies* no. 44 (4):519–544. doi: 10.1080/00220380801980855.

Groh, Sebastian. 2014. “The role of energy in development processes—The energy poverty penalty: Case study of Arequipa (Peru).” *Energy for Sustainable Development* no. 18 (0):83–99. doi: 10.1016/j.esd.2013.12.002.

Kerstenetzky, Celia Lessa, and Larissa Santos. 2009. “Poverty as Deprivation of Freedom: The Case of Vidigal Shantytown in Rio de Janeiro.” *Journal of Human Development and Capabilities* no. 10 (2):189. doi: 10.1080/19452820902940893.

Neff, Daniel. 2013. “Fuzzy set theoretic applications in poverty research.” *Policy and Society* no. 32 (4):319–331. doi: 10.1016/j.polsoc.2013.10.004.

Ningaye, Paul, TiomelaYemedjeu Alexi, and TakoutioFeudjio Virginie. 2013. “Multi-Poverty in Cameroon: A Structural Equation Modeling Approach.” *Social Indicators Research* no. 113 (1):159–181. doi: 10.1007/s11205-012-0087-8.

Norcia, Maurizio, Antonella Risotto, and Elisa Noci. 2012. “Measuring poverty through capabilities: preliminary results of a research in Italy.” *OIDA International Journal of Sustainable Development* no. 4 (8):39–46.

Parks, Sarah. 2012. “Divergent pathways of development: a comparative case study of human well-being in two Thai provinces.” *Environment and Planning C: Government and Policy* no. 30:891–909.

Roche, JoséManuel. 2013. “Monitoring Progress in Child Poverty Reduction: Methodological Insights and Illustration to the Case Study of Bangladesh.” *Social Indicators Research* no. 112 (2):363–390. doi: 10.1007/s11205-013-0252-8.

Roelen, Keetie, Franziska Gassmann, and Chris de Neubourg. 2010. “Child Poverty in Vietnam: Providing Insights Using a Country-Specific and Multidimensional Model.” *Social Indicators Research* no. 98 (1):129–145. doi: 10.1007/s11205-009-9522-x.

Santos, MariaEmma. 2013. “Tracking Poverty Reduction in Bhutan: Income Deprivation Alongside Deprivation in Other Sources of Happiness.” *Social Indicators Research* no. 112 (2):259–290. doi: 10.1007/s11205-013-0248-4.

Trani, Jean-Francois, Mario Biggeri, and Vincenzo Mauro. 2013. “The Multidimensionality of Child Poverty: Evidence from Afghanistan.” *Social Indicators Research* no. 112 (2):391–416. doi: 10.1007/s11205-013-0253-7.

Trani, Jean-François, and Tim I. Cannings. 2013. “Child Poverty in an Emergency and Conflict Context: A Multidimensional Profile and an Identification of the Poorest Children in Western Darfur.” *World Development* no. 48 (0):48–70. doi: 10.1016/j.worlddev.2013.03.005.

### Group iv. Poverty and Well-Being Assessment in Advanced Economies

Anand, Paul, Graham Hunter, Ian Carter, Keith Dowding, Francesco Guala, and Martin Van Hees. 2009. “The Development of Capability Indicators.” *Journal of Human Development and Capabilities* no. 10 (1):127. doi: 10.1080/14649880802675366.

Arndt, Christian, and Jürgen Volkert. 2011. “The Capability Approach: A Framework for Official German Poverty and Wealth Reports.” *Journal of Human Development and Capabilities* no. 12 (3):311–337. doi: 10.1080/19452829.2011.589248.

Bellani, Luna, Graham Hunter, and Paul Anand. 2013. “Multidimensional Welfare: Do Groups Vary in Their Priorities and Behaviours?*.” *Fiscal Studies* no. 34 (3):333–354. doi: 10.1111/j.1475-5890.2013.12009.x.

Beyazit, Eda. 2010. “Evaluating Social Justice in Transport: Lessons to be Learned from the Capability Approach.” *Transport Reviews* no. 31 (1):117–134. doi: 10.1080/01441647.2010.504900.

Binder, Martin, and Tom Broekel. 2011. “Applying a Non‐parametric Efficiency Analysis to Measure Conversion Efficiency in Great Britain.” *Journal of Human Development and Capabilities* no. 12 (2):261. doi: 10.1080/19452829.2011.571088.

Boarini, Romina, and Marco Mira D’Ercole. 2013. “Going beyond GDP: An OECD Perspective*.” *Fiscal Studies* no. 34 (3):289–314. doi: 10.1111/j.1475-5890.2013.12007.x.

Burchardt, Tania. 2009. “Agency Goals, Adaptation and Capability Sets.” *Journal of Human Development and Capabilities* no. 10 (1):3–19. doi: 10.1080/14649880802675044.

Burchardt, Tania, and Polly Vizard. 2011. “‘Operationalizing’ the Capability Approach as a Basis for Equality and Human Rights Monitoring in Twenty‐first‐century Britain.” *Journal of Human Development and Capabilities* no. 12 (1):91–119. doi: 10.1080/19452829.2011.541790.

Callander, Emily J., Deborah J. Schofield, and Rupendra N. Shrestha. 2012a. “Capacity for Freedom—Using a New Poverty Measure to Look at Regional Differences in Living Standards within Australia.” *Geographical Research* no. 50 (4):411–420. doi: 10.1111/j.1745-5871.2011.00748.x.

Callander, Emily J., Deborah J. Schofield, and Rupendra N. Shrestha. 2012b. “Towards a holistic understanding of poverty: A new multidimensional measure of poverty for Australia.” *Health Sociology Review* no. 21 (2):141–155. doi: 10.5172/hesr.2012.21.2.141.

Callander, EmilyJ, DeborahJ Schofield, and RupendraN Shrestha. 2012c. “Capacity for Freedom—A New Way of Measuring Poverty Amongst Australian Children.” *Child Indicators Research* no. 5 (1):179. doi: 10.1007/s12187-011-9122-6.

Clark, David, and David Hulme. 2010. “Poverty, time and vagueness: integrating the core poverty and chronic poverty frameworks.” *Cambridge Journal of Economics* no. 34 (2):347–366. doi: 10.1093/cje/ben046.

Clery, Elizabeth, Tiffany Tsang, and Polly Vizard. 2014. “The Children’s Measurement Framework: A new Indicator-Based Tool for Monitoring Children’s Equality and Human Rights.” *Child Indicators Research* no. 7 (2):321–349. doi: 10.1007/s12187-013-9224-4.

Gardoni, Paolo, and Colleen Murphy. 2014. “A Scale of Risk.” *Risk Analysis* no. 34 (7):1208–1227. doi: 10.1111/risa.12150.

Hofmann, Karen, Dominik Schori, and Thomas Abel. 2013. “Self-Reported Capabilities Among Young Male Adults in Switzerland: Translation and Psychometric Evaluation of a German, French and Italian Version of a Closed Survey Instrument.” *Social Indicators Research* no. 114 (2):723–738. doi: 10.1007/s11205-012-0170-1.

Jordan, Kirrily, Hannah Bulloch, and Geoff Buchanan. 2010. “Statistical equality and cultural difference in Indigenous wellbeing frameworks: A new expression of an enduring debate.” *Australian Journal of Social Issues* no. 45 (3):333–362,295-296.

Matsuyama, Jun, and Kenji Mori. 2011. “Freedom and achievement of well-being and adaptive dynamics of capabilities.” *Metroeconomica* no. 62 (3):494–511. doi: 10.1111/j.1467-999X.2011.04118.x.

Murphy, Colleen, and Paolo Gardoni. 2008. “The Acceptability and the Tolerability of Societal Risks: A Capabilities-based Approach.” *Science and Engineering Ethics* no. 14 (1):77–92. doi: 10.1007/s11948-007-9031-8.

Murphy, Colleen, and Paolo Gardoni. 2010. “Assessing capability instead of achieved functionings in risk analysis.” *Journal of Risk Research* no. 13 (2):145. doi: 10.1080/13669870903126259.

Peichl, Andreas, and Nico Pestel. 2013. “Multidimensional Well-Being at the Top: Evidence for Germany*.” *Fiscal Studies* no. 34 (3):355–371. doi: 10.1111/j.1475-5890.2013.12010.x.

Perrons, Diane. 2011. “Regional performance and inequality: linking economic and social development through a capabilities approach.” *Cambridge Journal of Regions, Economy and Society*. doi: 10.1093/cjres/rsr033.

Van Ootegem, Luc, and Elsy Verhofstadt. 2012. “Using Capabilities as an Alternative Indicator for Well-being.” *Social Indicators Research* no. 106 (1):133–152. doi: 10.1007/s11205-011-9799-4.

Waglé, Udaya R. 2008. “Multidimensional poverty: An alternative measurement approach for the United States?” *Social Science Research* no. 37 (2):559–580. doi: 10.1016/j.ssresearch.2007.06.013.

Wagle, Udaya R. 2009. “Capability Deprivation and Income Poverty in the United States, 1994 and 2004: Measurement Outcomes and Demographic Profiles.” *Social Indicators Research* no. 94 (3):509–533. doi: 10.1007/s11205-009-9446-5.

Wagle, Udaya R. 2014. “The Counting-Based Measurement of Multidimensional Poverty: The Focus on Economic Resources, Inner Capabilities, and Relational Resources in the United States.” *Social Indicators Research* no. 115 (1):223–240. doi: 10.1007/s11205-012-0216-4.

Wüst, Kirsten, and Jürgen Volkert. 2012. “Childhood and Capability Deprivation in Germany: A Quantitative Analysis Using German Socio-Economic Panel Data.” *Social Indicators Research* no. 106 (3):439–469. doi: 10.1007/s11205-011-9817-6.

### Group v. Deprivation of Disabled People

Rosano, Aldo, Federica Mancini, and Alessandro Solipaca. 2009. “Poverty in People with Disabilities: Indicators from the Capability Approach.” *Social Indicators Research* no. 94 (1):75–82. doi: 10.1007/s11205-008-9337-1.

### Group vi. Assessing Gender Inequalities

Addabbo, Tindara, Maria Laura Di Tommaso, and Anna Maccagnan. 2014. “Gender Differences in Italian Children’s Capabilities.” *Feminist Economics* no. 20 (2):90–121. doi: 10.1080/13545701.2013.844846.

Addabbo, Tindara, Diego Lanzi, and Antonella Picchio. 2010. “Gender Budgets: A Capability Approach.” *Journal of Human Development and Capabilities* no. 11 (4):479–501. doi: 10.1080/19452829.2010.520900.

Anand, Paul, and Cristina Santos. 2007. “Violent crime, gender inequalities and well-being: models based on a survey of individual capabilities and crime rates for England and Wales.” *Revue d’économie politique* no. 117:135-160.

Bérenger, Valérie, and Audrey Verdier‐Chouchane. 2011. “From the Relative Women Disadvantage Index to Women’s Quality‐of‐Life.” *Journal of Human Development and Capabilities* no. 12 (2):203–233. doi: 10.1080/19452829.2010.520893.

Di Tommaso, Maria L., Isilda Shima, Steinar Strøm, and Francesca Bettio. 2009. “As bad as it gets: Well-being deprivation of sexually exploited trafficked women.” *European Journal of Political Economy* no. 25 (2):143–162. doi: 10.1016/j.ejpoleco.2008.11.002.

Floro, Maria S., and Anant Pichetpongsa. 2010. “Gender, Work Intensity, and Well-Being of Thai Home-Based Workers.” *Feminist Economics* no. 16 (3):5–44. doi: 10.1080/13545701.2010.499657.

Gálvez-Muñoz, Lina, Mónica Domínguez-Serrano, Paula Rodríguez-Modroño, and Mauricio Matus-López. 2013. “Gender, Time Use, and Children’s and Adolescents’ Well-Being: Implications for Public Policies*.” *Fiscal Studies* no. 34 (3):373–389. doi: 10.1111/j.1475-5890.2013.12011.x.

### Group vii. Debating Policies

Agee, Mark D., and Thomas D. Crocker. 2013. “Operationalizing the capability approach to assessing well-being.” *The Journal of Socio*-*Economics* no. 46 (0):85. doi: 10.1016/j.socec.2013.07.003.

Burchi, Francesco, and Andrea Passacantilli. 2013. “Inequality in the monetary and functioning spaces: the case of Peru under the first Garcia government (1985–1990).” *Journal of International Development* no. 25 (3):340–361. doi: 10.1002/jid.1821.

Fahlén, Susanne. 2013. “Capabilities and Childbearing Intentions in Europe.” *European Societies* no. 15 (5):639–662. doi: 10.1080/14616696.2013.798018.

Hirvilammi, Tuuli, Senja Laakso, Michael Lettenmeier, and Satu Lähteenoja. 2013. “Studying Well-being and its Environmental Impacts: A Case Study of Minimum Income Receivers in Finland.” *Journal of Human Development and Capabilities* no. 14 (1):134–154. doi: 10.1080/19452829.2012.747490.

Hobson, Barbara, and Susanne Fahlén. 2009. “Competing Scenarios for European Fathers: Applying Sen’s Capabilities and Agency Framework to Work—Family Balance.” *The ANNALS of the American Academy of Political and Social Science* no. 624 (1):214–233. doi: 10.1177/0002716209334435.

Hobson, Barbara, Susanne Fahlén, and Judit Takács. 2011. “Agency and Capabilities to Achieve a Work–Life Balance: A Comparison of Sweden and Hungary.” *Social Politics: International Studies in Gender, State & Society* no. 18 (2):168–198. doi: 10.1093/sp/jxr007.

Kalfagianni, Agni. 2014. “Addressing the Global Sustainability Challenge: The Potential and Pitfalls of Private Governance from the Perspective of Human Capabilities.” *Journal of Business Ethics* no. 122 (2):307–320. doi: 10.1007/s10551-013-1747-6.

Reitinger, Claudia, Matthias Dumke, Mario Barosevcic, and Rafaela Hillerbrand. 2011. “A conceptual framework for impact assessment within SLCA.” *The International Journal of Life Cycle Assessment* no. 16 (4):380–388. doi: 10.1007/s11367-011-0265-y.

Renouard, Cecile. 2011. “Corporate Social Responsibility, Utilitarianism, and the Capabilities Approach.” *Journal of Business Ethics* no. 98 (1):85. doi: 10.1007/s10551-010-0536-8.

### Group viii. Education

Kelly, Anthony. 2012. “Sen and the art of educational maintenance: evidencing a capability, as opposed to an effectiveness, approach to schooling.” *Cambridge Journal of Education* no. 42 (3):283–296. doi: 10.1080/0305764X.2012.706255.

Maddox, Bryan, and Lucio Esposito. 2011. “Sufficiency Re-examined: A Capabilities Perspective on the Assessment of Functional Adult Literacy.” *The Journal of Development Studies* no. 47 (9):1315–1331. doi: 10.1080/00220388.2010.509788.

Maguire, Cindy, Corinne Donovan, Jacob Mishook, Genevieve de Gaillande, and Ivonne Garcia. 2012. “Choosing a life one has reason to value: the role of the arts in fostering capability development in four small urban high schools.” *Cambridge Journal of Education* no. 42 (3):367–390. doi: 10.1080/0305764X.2012.706258.

McLean, Monica, and Melanie Walker. 2011. “The possibilities for university-based public-good professional education: a case-study from South Africa based on the ‘capability approach’.” *Studies in Higher Education* no. 37 (5):585–601. doi: 10.1080/03075079.2010.531461.

Smith, Michèle, and Angeline M. Barrett. 2011. “Capabilities for learning to read: An investigation of social and economic effects for Grade 6 learners in Southern and East Africa.” *International Journal of Educational Development* no. 31 (1):23–36. doi: 10.1016/j.ijedudev.2010.06.006.

Tikly, Leon, and Angeline M. Barrett. 2011. “Social justice, capabilities and the quality of education in low income countries.” *International Journal of Educational Development* no. 31 (1):8. doi: 10.1016/j.ijedudev.2010.06.001.

Walker, Melanie. 2006. “Towards a capability‐based theory of social justice for education policy‐making.” *Journal of Education Policy* no. 21 (2):163–185. doi: 10.1080/02680930500500245.

Walker, Melanie. 2008. “A human capabilities framework for evaluating student learning.” *Teaching in Higher Education* no. 13 (4):477–487. doi: 10.1080/13562510802169764.

Walker, Melanie. 2012. “A capital or capabilities education narrative in a world of staggering inequalities?” *International Journal of Educational Development* no. 32 (3):384–393. doi: 10.1016/j.ijedudev.2011.09.003.

Young, Marion. 2009. “Basic Capabilities, Basic Learning Outcomes and Thresholds of Learning.” *Journal of Human Development and Capabilities* no. 10 (2):259–277. doi: 10.1080/19452820902941206.

### Group ix. Technology

Grunfeld, Helena, Sokleap Hak, and Tara Pin. 2011. “Understanding benefits realisation of iREACH from a capability approach perspective.” *Ethics and Information Technology* no. 13 (2):151–172. doi: 10.1007/s10676-011-9268-4.

Hatakka, Mathias, Annika Andersson, and Åke Grönlund. 2013. “Students’ use of one to one laptops: a capability approach analysis.” *Information Technology & People* no. 26 (1):94–112. doi: doi:10.1108/09593841311307169.

Hatakka, Mathias, and Jenny Lagsten. 2011. “The capability approach as a tool for development evaluation—analyzing students’ use of internet resources.” *Information Technology for Development* no. 18 (1):23–41. doi: 10.1080/02681102.2011.617722.

Kivunike, Florence Nameere, Love Ekenberg, Mats Danielson, and F. F. Tusubira. 2011. “Perceptions of the role of ICT on quality of life in rural communities in Uganda.” *Information Technology for Development* no. 17 (1):61–80. doi: 10.1080/02681102.2010.511698.

Kleine, Dorothea. 2010. “ICT4WHAT?—Using the choice framework to operationalise the capability approach to development.” *Journal of International Development* no. 22 (5):674–692. doi: 10.1002/jid.1719.

Kleine, Dorothea. 2011. “The capability approach and the ‘medium of choice’: steps towards conceptualising information and communication technologies for development.” *Ethics and Information Technology* no. 13 (2):119–130. doi: 10.1007/s10676-010-9251-5.

Kleine, Dorothea, Ann Light, and Maria-José Montero. 2012. “Signifiers of the life we value? Considering human development, technologies and Fair Trade from the perspective of the capabilities approach.” *Information Technology for Development* no. 18 (1):42–60. doi: 10.1080/02681102.2011.643208.

Mizohata, Sachie, and Raynald Jadoul. 2013. “Towards International and Interdisciplinary Research Collaboration for the Measurements of Quality of Life.” *Social Indicators Research* no. 111 (3):683–708. doi: 10.1007/s11205-012-0027-7.

Ojo, Adegboyega, Tomasz Janowski, and Johanna Awotwi. 2013. “Enabling development through governance and mobile technology.” *Government Information Quarterly* no. 30, Supplement 1 (0):S32-S45. doi: 10.1016/j.giq.2012.10.004.

### Group x. Health

Al-Janabi, Hareth, Terry Flynn, and Joanna Coast. 2012. “Development of a self-report measure of capability wellbeing for adults: the ICECAP-A.” *Quality of Life Research* no. 21 (1):167–176. doi: 10.1007/s11136-011-9927-2.

Callander, Emily J, Deborah J Schofield, and Rupendra N Shrestha. 2013a. “Chronic health conditions and poverty: a cross-sectional study using a multidimensional poverty measure.” *BMJ Open* no. 3 (11). doi: 10.1136/bmjopen-2013-003397.

Callander, Emily J., Deborah J. Schofield, and Rupendra N. Shrestha. 2013b. “Freedom poverty: A new tool to identify the multiple disadvantages affecting those with CVD.” *International Journal of Cardiology* no. 166 (2):321–326. doi: 10.1016/j.ijcard.2011.10.088.

Coast, Joanna, Terry N. Flynn, Lucy Natarajan, Kerry Sproston, Jane Lewis, Jordan J. Louviere, and Tim J. Peters. 2008. “Valuing the ICECAP capability index for older people.” *Social Science & Medicine* no. 67 (5):874–882. doi: 10.1016/j.socscimed.2008.05.015.

Couzner, Leah, J. Ratcliffe, L. Lester, T. Flynn, and M. Crotty. 2013. “Measuring and valuing quality of life for public health research: application of the ICECAP-O capability index in the Australian general population.” *International Journal of Public Health* no. 58 (3):367–376. doi: 10.1007/s00038-012-0407-4.

Davis, Jennifer, Teresa Liu-Ambrose, ChrisG Richardson, and Stirling Bryan. 2013. “A comparison of the ICECAP-O with EQ-5D in a falls prevention clinical setting: are they complements or substitutes?” *Quality of Life Research* no. 22 (5):969–977. doi: 10.1007/s11136-012-0225-4.

Ferrer, Robert L., and Alejandra Varela Carrasco. 2010. “Capability and Clinical Success.” *The Annals of Family Medicine* no. 8 (5):454–460. doi: 10.1370/afm.1163.

Ferrer, Robert L., Inez Cruz, Sandra Burge, Bryan Bayles, and Martha I. Castilla. 2014. “Measuring Capability for Healthy Diet and Physical Activity.” *The Annals of Family Medicine* no. 12 (1):46–56. doi: 10.1370/afm.1580.

Grewal, Ini, Jane Lewis, Terry Flynn, Jackie Brown, John Bond, and Joanna Coast. 2006. “Developing attributes for a generic quality of life measure for older people: Preferences or capabilities?” *Social Science & Medicine* no. 62 (8):1891–1901. doi: 10.1016/j.socscimed.2005.08.023.

Lewis, Ferdinand. 2012a. “Auditing Capability and Active Living in the Built Environment.” *Journal of Human Development and Capabilities* no. 13 (2):295–315. doi: 10.1080/19452829.2011.645028.

Lewis, Ferdinand. 2012b. “Toward a general model of built environment audits.” *Planning Theory* no. 11 (1):44–65. doi: 10.1177/1473095211408056.

Mabsout, Ramzi. 2011. “Capability and Health Functioning in Ethiopian Households.” *Social Indicators Research* no. 101 (3):359–389. doi: 10.1007/s11205-010-9661-0.

McAllister, Marion, Graham Dunn, Katherine Payne, Linda Davies, and Chris Todd. 2012. “Patient empowerment: The need to consider it as a measurable patient-reported outcome for chronic conditions.” *BMC Health Services Research* no. 12 (1):157.

Mitchell, Paul M., Tracy E. Roberts, Pelham M. Barton, Beth S. Pollard, and Joanna Coast. 2013. “Predicting the ICECAP-O Capability Index from the WOMAC Osteoarthritis Index: Is Mapping onto Capability from Condition-Specific Health Status Questionnaires Feasible?” *Medical Decision Making* no. 33 (4):547–557. doi: 10.1177/0272989x12475092.

Mitra, Sophie, Kris Jones, Brandon Vick, David Brown, Eileen McGinn, and MaryJane Alexander. 2013. “Implementing a Multidimensional Poverty Measure Using Mixed Methods and a Participatory Framework.” *Social Indicators Research* no. 110 (3):1061–1081. doi: 10.1007/s11205-011-9972-9.

Netten, Ann, Peter Burge, Juliette Malley, Dimitris Potoglou, Ann-Marie Towers, John Brazier, Terry Flynn, and Julien Forder. 2012. “Outcomes of social care for adults: developing a preference-weighted measure.” *Health Technology Assessment* no. 16 (16):1–166.

Nikiema, Beatrice, Slim Haddad, and Louise Potvin. 2012. “Measuring women’s perceived ability to overcome barriers to healthcare seeking in Burkina Faso.” *BMC Public Health* no. 12 (1):147.

Ratcliffe, Julie, Laurence H. Lester, Leah Couzner, and Maria Crotty. 2013. “An assessment of the relationship between informal caring and quality of life in older community-dwelling adults—more positives than negatives?” *Health & Social Care in the Community* no. 21 (1):35–46. doi: 10.1111/j.1365-2524.2012.01085.x.

Simon, Judit, Paul Anand, Alastair Gray, Jorun Rugkåsa, Ksenija Yeeles, and Tom Burns. 2013. “Operationalising the capability approach for outcome measurement in mental health research.” *Social Science & Medicine* no. 98 (0):187–196. doi: 10.1016/j.socscimed.2013.09.019.
